# An improved spectral estimation method based on color perception features of mobile phone camera

**DOI:** 10.3389/fnins.2022.1031505

**Published:** 2022-10-19

**Authors:** Duan Liu, Xinwei Wu, Jinxing Liang, Tengfeng Wang, Xiaoxia Wan

**Affiliations:** ^1^Research Center of Graphic Communication, Printing and Packaging, Wuhan University, Wuhan, China; ^2^School of Computer Science and Artificial Intelligence, Wuhan Textile University, Wuhan, China; ^3^Hubei Province Engineering Technical Center for Digitization and Virtual Reproduction of Color Information of Cultural Relics, Wuhan, China

**Keywords:** spectral estimation, spectral reflectance, mobile phone camera, color perception feature, CIELAB uniform color space, spectral imaging, sample weighting, raw response

## Abstract

We use the mobile phone camera as a new spectral imaging device to obtain raw responses of samples for spectral estimation and propose an improved sequential adaptive weighted spectral estimation method. First, we verify the linearity of the raw response of the cell phone camera and investigate its feasibility for spectral estimation experiments. Then, we propose a sequential adaptive spectral estimation method based on the CIE1976 L*a*b* (CIELAB) uniform color space color perception feature. The first stage of the method is to weight the training samples and perform the first spectral reflectance estimation by considering the Lab color space color perception features differences between samples, and the second stage is to adaptively select the locally optimal training samples and weight them by the first estimated root mean square error (RMSE), and perform the second spectral reconstruction. The novelty of the method is to weight the samples by using the sample in CIELAB uniform color space perception features to more accurately characterize the color difference. By comparing with several existing methods, the results show that the method has the best performance in both spectral error and chromaticity error. Finally, we apply this weighting strategy based on the CIELAB color space color perception feature to the existing method, and the spectral estimation performance is greatly improved compared with that before the application, which proves the effectiveness of this weighting method.

## Introduction

The surface spectral reflectance is known as the fingerprint of object colors (Wang et al., 2020) and can characterize colors more accurately than RGB trichromatic information, enabling the replication and reproduction of color information. Therefore, obtaining accurate spectral information through spectral imaging is a hot topic in color science research. This makes spectral imaging technology widely used in many fields, such as heritage conservation, remote sensing mapping, skin disease diagnosis, printing color management, food safety monitoring, material non-destructive testing, etc. (Del Pozo et al., 2017; Liang and Wan, 2017; Rateni et al., 2017; Huang et al., 2021; Liang et al., 2021; Tominaga et al., 2022).

In the past decades, a large number of color scientists and research institutions have constructed a large number of spectral imaging systems that consist of monochromatic or multicolor cameras, combined with filter sets, and multiple light sources (Liang and Wan, 2017). However, due to the low imaging efficiency, low spatial resolution, and high system construction cost of such multichannel spectral imaging systems, color scientist Murakami started to study the method of estimating spectral reflectance from single RGB images of digital cameras in 2002 (Murakami et al., 2002).

The spectral reflectance estimation method based on digital camera responses is to solve the ill-posed inverse problem mathematically and estimate the high-dimensional spectral reflectance from the low-dimensional camera responses (Xiao et al., 2019). Compared with multichannel spectral imaging methods, this method has the advantages of fast imaging speed, high spatial resolution, avoidance of geometric distortion, and good economy (Liang et al., 2019) which makes professional SLR digital cameras gradually become the new spectral imaging devices.

Since Kyocera released the first camera-equipped mobile phone in 1999 (Hussain and Bowden, 2021), camera technology on mobile phones has developed rapidly. Today, the mainstream high-end cell phone camera has a resolution of at least 64 megapixels. In terms of hardware, super large-area CMOS, Optical Image Stabilizer, large aperture, Phase Auto Focus, 4-in-1 Super Pixel, and many other new technologies have been popularized. The software adds features like AI portrait, super night photo, time-lapse mode, live photo, slow motion video in 120 fps, etc. Hardware and software upgrades have greatly improved the imaging capability of cameras on the cell phone, and its gap with professional digital cameras is gradually being smoothed out, making mobile phones gradually become a daily shooting tool for most people, and also become the imaging instrument for many scientific studies, used to replace the heavy, expensive professional digital cameras (Kim et al., 2019; Hussain and Bowden, 2021; Stuart et al., 2021; Liang et al., 2022; Tominaga et al., 2022).

The spectral estimation methods based on a single RGB image mainly include the Wiener method, Infinite-dimensional model method, pseudo-inverse method, R matrix method, principal component analysis (PCA) method, kernel method, and so on. Since the camera sensitivity required by the Wiener method and the infinite-dimensional model is difficult to obtain directly (Tominaga et al., 2021), and the indirect method of estimating sensitivity increases the error, these two methods are less applied. In contrast, methods such as pseudo-inverse estimation, R-matrix, and PCA have the advantages of no prior data, simple processes, and small computational, but the spectral estimation accuracy of these methods is relatively low. To improve the estimation accuracy, a large number of optimization methods have been proposed. Connah and Hardeberg (2005) introduced the polynomial model to three-channel and multi-channel spectral imaging systems. Heikkinen et al. (2007) proposed an application of the regularization framework to estimate spectral reflectance from digital camera responses. Shen et al. (2010) proposed a partial least squares-based spectral estimation method that improves on the least square regression method. Xiao et al. (2016) combined the polynomial model with the principal component analysis (PCA) method in eigenvector space and applied it to skin color detection (Xiao et al., 2016). These improved methods have achieved some improvement in estimation accuracy, but they are all based on global training, which poses a limitation on the performance of the method. Subsequent studies in which training samples were optimally selected and weighted emerged. Cao proposed a spectral reflectance estimation method for local linear estimation with sample selection optimization (Cao et al., 2017). Zhang et al. (2017) proposed a spectral estimation method based on local sample selection based on CIEXYZ color space color difference under multiple light sources. Shen proposed a scanner-based local training sample weighted spectral estimation method (Shen and Xin, 2006). Liang and Wan (2017) proposed a local inverse distance weighted linear regression method for spectral estimation from camera response. Amiri and Amirshahi (2014) also studied the weighted non-linear regression models in the form of global weighting. These methods weight the samples by calculating the weight matrix based on the Euclidean distance between the training samples and the test samples in the color space, and although they have better spectral estimation accuracy than previous methods, they still have certain shortcomings because the RGB color space is device-dependent and not a uniform color space, which has a large difference from the color difference perceived by the human eye, and the above methods perform sample selection and weighting by color difference. The method ignores the spectral differences between samples. Wang proposed a two-time sequence weighting method considering the chromaticity and spectral error at the same time, and adaptively optimized the sample selection, which achieved good results (Wang et al., 2020).

Through the development of spectral estimation methods, we can conclude that the optimal selection of samples is extremely important, and how the samples are optimally selected and weighted largely determines the estimation accuracy of the estimation method. The more similar the training and testing samples are in the color space, the better the estimation accuracy, so samples with similar colors should be selected for training as much as possible (Liang and Wan, 2017; Liang et al., 2022). At the same time, the chromaticity difference between the training and test samples is measured precisely, and different weights are assigned to obtain better estimation accuracy.

Meanwhile, we note that the existing methods using Euclidean distance or chromaticity vector angle in color space to measure the difference between training and testing samples are still not accurate. If multiple training samples have the same Euclidean distance to one test sample, but each training sample does not have the same color perception features as the target sample, the existing methods still assign the same weight to these training samples, which will produce an error and the phenomenon of Metamerism. This means that we can measure the color differences between samples more accurately if we take the differences in color perception features such as lightness, hue, and chroma into account.

In this paper, we explore new spectral imaging devices that use cell phone camera raw responses as a data source. We propose an improved sequential adaptive weighted spectral estimation method based on the color perception features of CILAB uniform color space. The novelty of the study is mainly in two aspects: (1) Using a cell phone camera to replace a professional digital camera as an imaging device, the linearity of the cell phone camera raw responses and its application to spectral estimation are verified to be feasible. (2) Using three perceptual features of CIE1976 L*a*b* (CIELAB) uniform color space for local sample selection and calculation of the weighting matrix to achieve a more accurate measure of color differences between samples. By comparing the proposed method with the existing method in a 10 times 10-fold cross-validation, all using raw responses output from the same cell phone, the experimental results show that the proposed method has the best performance in two aspects of spectral error and chromaticity error in four metrics. Finally, we apply the color perception features weighting strategy of the proposed method to the existing method, and the accuracy is significantly improved compared with the original method.

## Imaging model

Although the internal space of cell phones is small and their camera components are different from professional digital cameras, the imaging principle is still the same as that of digital cameras. The light emitted from the light source, after reflecting from the surface of the object, passes through the camera lens set and is converted by CMOS for optoelectric conversion to generate raw response signals, after processing by ISP chip, the mobile phone camera output the photo. So the three-channel response *y*_*i*_ of the camera is determined by the spectral power distribution *l*(λ) of the light source, the reflectance *r*(λ) of the object surface, the sensitivity function *m*(λ) of the cell phone camera system and the system noise *n*_*i*_ together, and we can write the imaging model of the cell phone camera as the following integral calculation process of Equation (1).


(1) $$y_{i}=\int_{\varphi }l\,\left(\lambda \right)\,r\,\left(\lambda \right)\,m\,\left(\lambda \right)\,d\lambda +n_{i}$$

Where the subscript i denotes the three channels of the camera, φ denotes the wavelength range of the visible spectrum. If we assume that the noise *n*_*i*_ = 0, for mathematical simplicity, Equation 1 can be written as the following matrix equation, as in Equation (2).


(2) y=M⁢r

Where *y* denotes the camera response vector, *M* denotes the spectral sensitivity matrix of the whole system including the spectral power distribution of the light source, the sensitivity function of the cell phone camera, and *r* is the surface spectral reflectance of the target object.

## Proposed method

Based on the imaging model of the camera, we can divide the spectral estimation method into 2 steps: the first step is to calculate the spectral conversion matrix by training samples. The training sample spectral reflectance $\tilde{r}$ is obtained by measurement, and the camera three-channel response value *y* is extracted from the raw file, and the conversion matrix *M* is calculated by the pseudo-inverse method, as in Equation (3).


(3) $$\tilde{r}=M\,y$$

The second step is to calculate the spectral reflectance of the target sample using the conversion matrix *M*. The high-dimensional spectral reflectance *R* of the target sample is estimated from the known three-channel response *Y* of the target sample, as in Equation (4).


(4) R=M⁢Y

The methods for calculating the conversion matrix *M*, as described previously, mainly include: the pseudo-inverse method, PCA, and other methods, and the calculation in this paper use the pseudo-inverse method, as shown in Equation (5).


(5) $$M=\left(R\,Y^{T}\right)\,{\left(Y\,Y^{T}\right)}^{-1}$$

Where the superscript T denotes matrix transpose and the superscript -1 denotes matrix inverse operation.

### Extraction and verification of cell phone camera raw response

JPG and HEIF format photos are processed and compressed by the cell phone ISP processor. To ensure that raw response can be obtained from the cell phone camera, we set the cell phone to output the raw format file and then compared them with the JPG format pictures of the same scene. Under the same light source conditions, the Xrite ColorChecker CLASSIC color chart (hereafter referred to as CC chart) was photographed using five cell phones, and the RGB three channel values of the grayscale in the fourth row of the CC chart were extracted separately to check the raw response linearity of different models and different brands of cell phone cameras (Liang et al., 2019) the results show that the cell phone camera raw response has good linearity. The experimental steps and results are detailed in “Imaging conditions and raw response extraction” and “Verification of cell phone camera raw response,” respectively.

Further, to verify the effect of the difference between the raw response of the cell phone camera and the professional digital camera on the spectral estimation, the above five cell phones were photographed with the Xrite ColorChecker SG chart (hereafter referred to as CCSG color chart) under the same light source conditions as described above, and 140 color blocks of the color charts were taken as the sample set, while the raw response output from a Nikon D3x professional digital camera was also used as a comparison benchmark. All six devices were used for spectral estimation experiments in both OLS and modified ALWLR methods, and the detailed experimental procedures and results are described in “Imaging conditions and raw response extraction” and “Verification of cell phone camera raw response,” respectively.

### The proposed method

**FIGURE 1:**
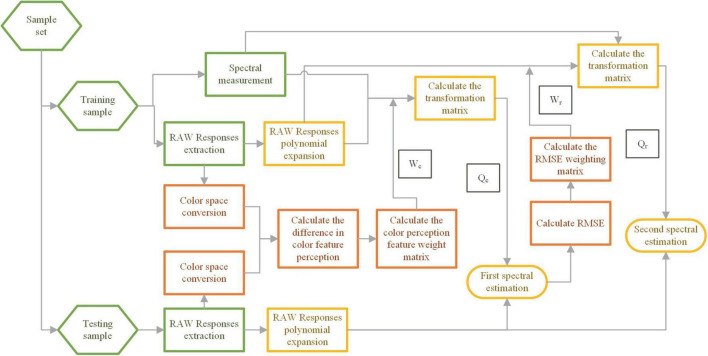
Flowchart of the proposed method.

The proposed method is based on the classical pseudo-inverse method for improvement. It uses two sequential adaptive sample weighting and optimal selection to improve performance. Based on the extraction of raw response data from cell phone cameras, the main process of the method is divided into the following five steps: The first step is to perform the color space conversion of the three-channel RGB values of the samples and the calculation of the color perception features differences in CIELAB uniform color space. The second step is to construct a color difference weight matrix *W*_*c*_ using the color perception feature differences and weighting the training samples. The third step is to calculate the transformation matrix *Q*_*c*_ using the weighted test sample raw response and spectral data, and then performing the first spectral estimation calculation using *Q*_*c*_. The fourth step is to calculate the root mean square error (RMSE) of the results of the first spectral estimation, to construct the spectral difference weighting matrix *W*_*r*_, and to select and weight the training samples for the second time. The fifth step uses the *W*_*r*_ matrix to weight the training samples and calculate the conversion matrix *Q*_*r*_, the second spectral estimation of the test samples is performed to obtain the spectral reflectance results. Figure 1 shows the flow chart of the method.

#### Color space conversion and color perception feature differences calculation

The RGB three-channel values of each sample are obtained from the cell phone camera raw response by the method described in “Extraction and verification of cell phone camera raw response,” and at this time the response RGB color space is related to the imaging device, and we choose the CIELAB uniform color space as the color space for calculating the color difference. First, the RGB stimulus values of the training samples are converted to CIELAB color space, and the color space conversion matrix *T* is obtained by the least square method, and *T* is calculated as Equation (6).


(6) $$T=\left(L_{t\,r\,a\,i\,n}\,D_{t\,r\,a\,i\,n}^{T}\right)\,{\left(D_{t\,r\,a\,i\,n}\,D_{t\,r\,a\,i\,n}^{T}\right)}^{-1}$$

*D_train_* denotes the response of the training sample; *L_train_* denotes the CIELAB value matrix of the training sample, and the superscript ‘T’ denotes the matrix transpose; the superscript ‘–1’ denotes the pseudo-inverse operations. Then we use this transformation matrix *T* to transform the camera RAW responses of the test samples into the CIELAB color space as in Equation (7).


(7) $$L_{t\,e\,s\,t}=T\,D_{t\,e\,s\,t}$$

*D_test_* denotes the raw response matrix of the target sample and *L_test_* denotes the color matrix of the test sample transformed to CIELAB color space.

The metrics of sample color differences in existing studies are mainly divided into 2 categories: one category is to use the color difference in color space, which is the Euclidean distance to measure the color difference between samples (Liang and Wan, 2017), and this method selects training samples with a similar color to the test samples by the color difference in a certain color space while giving different weights to different training samples to achieve local sample selection and weighting, which has the advantage of computational simplicity. Another category is to use chromaticity vector angle to calculate the angular difference between sample color vectors in some color space for sample selection and weighting (Wang et al., 2020). The advantage of this method is that a more uniform CIEXYZ uniform color space is used, and the color differences are more consistent with human eye perception, but the above two types of methods still have the problem that the measure of sample color differences is not accurate enough because this will enable samples with the same or similar Euclidean distance to obtain almost the same weight, while the actual situation is that there may be large differences in color perception features of the samples, which is the reason for the Metamerism phenomenon in this type of methods; and the chromaticity vector angle apparently has the same problem of being unable to characterize and measure the color perception features, which is still not accurate enough. Therefore, this study proposes to use three color perception feature measures of CIELAB uniform color space for sample color difference. In the CIELAB uniform color space, we assume that the color values of the training and test samples are ($L_{\mathrm{trian}}^{*},a_{\mathrm{trian}}^{*},b_{\mathrm{trian}}^{*}$) and ($L_{\mathrm{test}}^{*}$, $,a_{\mathrm{test}}^{*}$,$b_{\mathrm{test}}^{*}$), then the color difference $\Delta \,E_{\mathrm{ab}}^{*}$ between them can be calculated by Euclidean distance in the color space, as in Equation (8) below.


(8) $$\Delta \,E_{a\,b}^{*}=\sqrt{{\left(L_{t\,r\,i\,a\,n}^{*}-L_{t\,e\,s\,t}^{*}\right)}^{2}+{\left(a_{t\,r\,i\,a\,n}^{*}-a_{t\,e\,s\,t}^{*}\right)}^{2}+{\left(b_{t\,r\,i\,a\,n}^{*}-b_{t\,e\,s\,t}^{*}\right)}^{2}}$$

Also, the color difference $\Delta \,E_{\mathrm{ab}}^{*}$ can be expressed in terms of the differences in the color perception feature measures of the samples: lightness difference ΔL*, hue difference ΔH*, and chroma difference ΔC*, as in Equation (9).


(9) $$\Delta \,E_{a\,b}^{*}=\sqrt{{\left(\Delta \,L^{*}\right)}^{2}+{\left(\Delta \,H_{a\,b}^{*}\right)}^{2}+{\left(\Delta \,C_{a\,b}^{*}\right)}^{2}}$$

The color perception features ΔL*, $\Delta \,H_{\mathrm{ab}}^{*}$, and $\Delta \,C_{\mathrm{ab}}^{*}$ are calculated as Equations (10), (11), and (12).


(10) $$\Delta \,L^{*}=L_{t\,r\,a\,i\,n}^{*}-L_{t\,e\,s\,t}^{*}$$

(11) $$\Delta \,H_{a\,b}^{*}=\sqrt{{\left(\Delta \,E_{a\,b}^{*}\right)}^{2}-{\left(\Delta \,L^{*}\right)}^{2}-{\left(\Delta \,C^{*}\right)}^{2}}$$

(12) $$\Delta \,C_{a\,b}^{*}=\left|\sqrt{{\left(a_{t\,r\,a\,i\,n}^{*}\right)}^{2}+{\left(b_{t\,r\,a\,i\,n}^{*}\right)}^{2}}-\sqrt{{\left(a_{t\,e\,s\,t}^{*}\right)}^{2}+{\left(b_{t\,e\,s\,t}^{*}\right)}^{2}}\right|$$

#### Calculating the color perception feature weighting matrix

We use the ΔL*, $\Delta \,H_{\mathrm{ab}}^{*}$, and $\Delta \,C_{\mathrm{ab}}^{*}$ as our parameters for the first weighting matrix of the training samples, which will make the difference of any color perceptual feature directly affect the value of the sample weight, and these differences may not be expressed in the color difference of the Euclidean distance so that the color difference can be measured more precisely. The smaller the value means the smaller the difference in visual perception, we use the inverse of the sum of ΔL*, $\Delta \,H_{\mathrm{ab}}^{*}$, and $\Delta \,C_{\mathrm{ab}}^{*}$ to calculate the color perceptual feature weighting matrix *W*_*C*_, and the weight of the *i* th training sample *W*_*i*_ is calculated as in Equation (13).


(13) $$W_{i}=\frac{1}{\left|\Delta \,L_{i}^{*}\right|+\left|\Delta \,H_{i}^{*}\right|+\left|\Delta \,C_{i}^{*}\right|+\alpha },i\in \left\{1,2,\cdots ,k\right\}$$

To make the denominator non-zero, we introduce a very small value α. In this study, α = 0.0001 is taken. *k* is the total number of training samples. Then the weights of all training samples are arranged in descending order and converted into a diagonal matrix to form the color perception feature weighting matrix *W*_*C*_, as shown in Equation (14).


(14) WC=[W10⁢⋯00⋮W2⁢  00⁢⋱⋮00⋯⁢     0Wi]k*k

#### The first spectral estimation

Before the first spectral estimation, we performed a polynomial expansion of the cell phone camera raw response, and in “mobile phone raw response spectral estimation performance validation” we tested the effect of the number of polynomial terms on the estimation accuracy, and finally chose 18 terms to avoid the overfitting problem caused by too many terms. The expanded form of 18 terms is shown in Equation (15).


(15) $$y=\left[1\,r\,g\,b\,r\,g\,r\,b\,g\,b\,r^{2}\,g^{2}\,b^{2}\,r^{2}\,g\,r^{2}\,b\,g^{2}\,b\,g^{2}\,r\,b^{2}\,g\,g\,b^{2}\,r^{3}\,g^{3}\right]$$

After polynomial expansion, the weighted spectral transition matrix is calculated as Equation 16 according to Equation (16).


(16) $$Q_{C}=\left({\tilde{R}}_{t\,r\,a\,i\,n}\,{\tilde{Y}}_{t\,r\,a\,i\,n,e\,x\,p}^{T}\right)\,{\left({\tilde{Y}}_{t\,r\,a\,i\,n,e\,x\,p}\,{\tilde{Y}}_{t\,r\,a\,i\,n,e\,x\,p}^{T}\right)}^{-1}$$

Where ${\tilde{R}}_{\mathrm{train}}=W_{C}\,R_{\mathrm{train}}$,${\tilde{Y}}_{\mathrm{train},\exp}=W_{C}\,Y_{\mathrm{train}}$, ${\tilde{R}}_{\mathrm{train}}$ denotes the weighted training sample spectral reflectance matrix, and ${\tilde{Y}}_{\mathrm{train},\exp}^{T}$ denotes the weighted polynomial expansion matrix of the training sample. Finally, the transformation matrix *Q*_*C*_ is used to estimate the spectral reflectance of the test samples as in Equation (17).


(17) $${\hat{r}}_{t\,e\,s\,t}=Q_{C}\,y_{t\,e\,s\,t,e\,x\,p}$$

where ${\hat{r}}_{\mathrm{test}}$ denotes the estimated spectral reflectance of the test sample, and *y_test,exp_* denotes the polynomially expanded raw response matrix of the test sample.

#### Sample weighting and optimal selection based on root mean square error

After the spectral reflectance results are obtained by the previous step, the RMSE of spectral reflectance is calculated for the second weighting and selection of the training samples, and the RMSE of the spectral is calculated as Equation (18).


(18) $$R\,M\,S\,E_{j}=\sqrt{\frac{1}{n}\,{\left({\hat{r}}_{t\,e\,s\,t}-r_{j}\right)}^{T}\,\left({\hat{r}}_{t\,e\,s\,t}-r_{j}\right)},j\in \left\{1,2,\cdots ,k\right\}$$

n denotes the sampling resolution of visible light wavelength range, the range in this study is 400–700 nm, and the spectral sampling resolution is 10 nm, so n is 31 in this study. The training samples are arranged in ascending order according to the RMSE of the samples, the smaller the RMSE means the smaller the spectral difference between training samples and testing samples, in this step we use the inverse of the RMSE to construct the weight matrix, the weight *W*_*j*_ of each training sample is calculated as in Equation (19).


(19) $$W_{j}=\frac{1}{R\,M\,S\,E_{j}+\beta }$$

β is similar to α in Equation (12) and is a very small constant that makes the denominator non-zero, taking β = 0.0001. Then the first L local samples are selected by sample sorting according to *W*_*j*_ and the second diagonal weight matrix *W*_*R*_ is constructed in the same way as the Equation (13) in Equation (20).


(20) WR=[W10⁢⋯00⋮W2⁢  00⁢⋱⋮00⋯⁢     0Wj]j*j

#### The second spectral estimation

The response values of the training and testing samples are weighted in the same way using *W*_*R*_ to update to obtain *Y_train_* and *R*_*train*_, and then the new transformation matrix *Q*_*R*_ is calculated using Equation (16). Finally, the spectral reflectance of the testing sample ${\hat{r}}_{\mathrm{test}}^{{}^{\prime }}$ is calculated as in Equation (21).


(21) $${\hat{r}}_{t\,e\,s\,t}^{\prime }=Q_{R}\,y_{t\,e\,s\,t,e\,x\,p}$$

## Experiment

To verify the usability of cell phone camera raw response. First, linear verification experiments and spectral estimation performance experiments of cell phone camera raw response were conducted. Then, the optimization method of sequence adaptive weighted spectral estimation based on color perception features proposed in this paper has experimented with the existing methods. Finally, the strategy based on color perception features weighting is applied and compared with the existing methods. The raw responses of the cell phone camera for these experiments are obtained in the same experimental environment.

### Imaging conditions and raw response extraction

**FIGURE 2:**
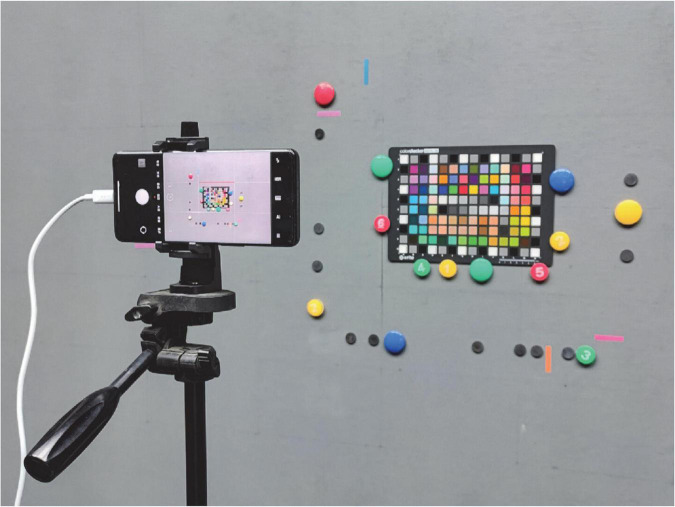
Mobile phone imaging scene.

**FIGURE 3:**
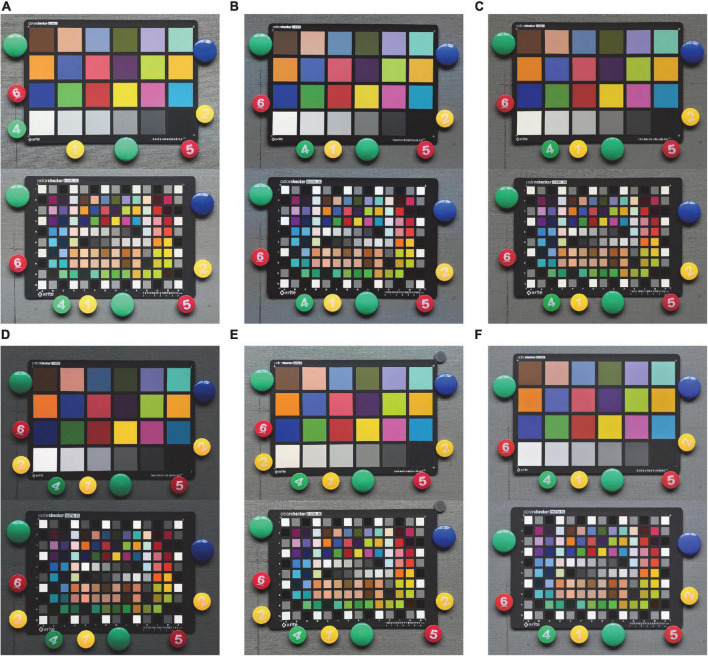
JPG pictures of the Xrite CC chart and SG chart were taken by cell phones, photos of the CC chart are arranged on the top, SG color chart is arranged on the bottom: **(A)** Huawei Mate10, **(B)** Meizu 17Pro, **(C)** Xiaomi12S Ultra, **(D)** iPhone 8, **(E)** iPhone 12, and **(F)** Nikon D3x.

To avoid interference of imaging by cluttered light, the experiments were conducted in a completely shaded laboratory with neutral gray walls. The experimental lighting source was a fluorescent lamp set with a color temperature of about 6000 K. The color chart was fixed on a vertical wall by magnets, and the height of the color chart was adjusted so that the fluorescent lamp irradiated to chart at an angle of 45 degrees, and the cell phone was set on a tripod and fixed by a holder to avoid shaking. The horizontal height of the cell phone was kept the same as the center point of the color card. The cell phone imaging scene is shown in Figure 2, and some of the pictures of the color chart taken by five cell phones and one DSLR camera are shown in Figure 3. These photos were taken in different formats (including DNG raw files and JPG post-processed photos).

**FIGURE 4:**
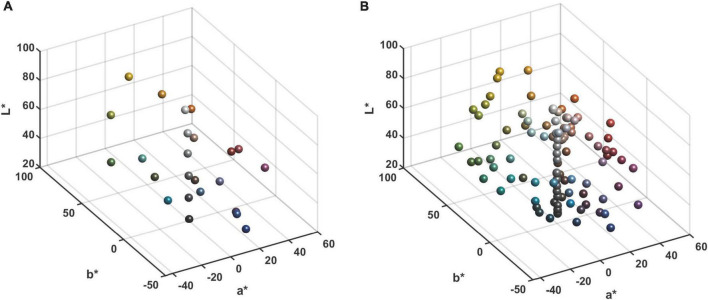
Distribution of the Xrite ColorChecker chart samples in CIELAB color space: **(A)** The CC chart. **(B)** The SG chart.

The experiments were conducted using the Xrite ColorChecker CLASSIC color chart (hereafter referred to as CC chart), which contains six grayscale gradient color blocks in the last row. The distribution of the color blocks in the CIELAB color space for the CC and SG color charts is depicted in Figure 4.

The shooting phones contain three Android phones and two Apple phones, while the Nikon D3x camera is used for comparison. The main information and CMOS parameters of the 6 shooting devices are shown in Table 1. Most of the phones have built-in camera apps for taking photos, and for phones where the camera app does not support the raw format output function, Lightroom software was used to take photos to obtain the raw response in DNG format 111^1^, and the raw file of the Nikon D3x camera was in NEF format F2^2^.

**TABLE 1:** Six imaging devices information.

Mobile phone	Release date	CMOS	CMOS size	Aperture	Resolution	CFA pattern	Quad-bayer or not
Huawei Mate10	October 16, 2017	SONY IMX286	1/2.9″	F1.6	12 MP	rggb	No
Meizu 17 Pro	May 8, 2020	SONY IMX686	1/1.73″	F1.8	64 MP	rggb	Yes
Xiaomi 12S Ultra	July 8, 2022	SONY IMX989	1″	F1.9	50 MP	rggb	Yes
Apple iPhone 8	September. 12, 2017	Unknown	Unknown	F1.8	12 MP	rggb	No
Apple iPhone 12	October 23, 2020	Unknown	Unknown	F1.6	12 MP	rggb	No
Nikon D3x	December. 1, 2008	Unknown	Full frame (35.9*24 mm)	Depends on lens	24 MP	rggb	No

The data content of the raw file is mainly the four-channel response of the CMOS Bayer filter array. We wrote a raw file batch processing program based on the open source software dcraw in MATLAB which contains three main steps: linear normalization of the response value, white balance recovery and demosaicing, and outputs a linear uncompressed three-channel TIFF image after processing. Subsequently, the specified area is intercepted to obtain the RGB three-channel average value. Through the above steps, we convert the camera raw response to three-channel RGB value and complete the extraction of raw response color information, and the processing of raw data in the above are linear transformations (Rob, 2014).

Since the main camera of the phone is usually the best CMOS on the phone with better imaging capability and better optical components, the shots are taken using the main camera of the phone. At the same time, the focal length and aperture of cell phone CMOS are usually fixed, and the aperture size of each cell phone main camera is not the same, so we take a fixed ISO, adjust the shutter time of each device to get the right camera response (D3x uses separate shooting parameters), and try to make the maximum value of the three channels of the white color block between 230 and 245 for each photo to avoid overexposure or underexposure to ensure a good dynamic range.

The spectral reflectance of each color block was measured by Xrite CI64 spectrophotometer and Color iControl software. The spectral reflectance was taken in the range of 400–700 nm with a wavelength interval of 10 nm and a measurement aperture of 6 mm.

### Evaluation metrics

The spectral estimation methods were all run using a ten times ten-fold cross-validation. The method randomly aliquots the samples into 10 groups, with a training sample of 9 groups and a test sample of 1 group, and cycles them 10 times to ensure the stability of the estimation results. The evaluation of the accuracy of the spectral estimation method includes four indicators: (1) the root-mean-square error of the spectrum is calculated as the average difference in the values of the spectral curves at each wavelength, and the calculation formula is the same as the Equation (18) in 3.2.4, the value range is 0–1, the smaller the value indicates the smaller the error; (2) The spectral goodness-of-fit coefficient measures the similarity of the shape of the spectral curves of the two samples by the angular cosine difference of their spectral curves, and the result ranges from 0 to 1. The closer to 1 means that the estimated spectral curve fits the actual spectral curve better, and the calculation formula is as in Equation (21).


(22) $$G\,F\,C=\frac{{\hat{r}}^{T}\,r}{||{\hat{r}}^{T}\,\hat{r}||\,\;||r^{T}\,r||}$$

(3) The CIELAB color difference is $\Delta \,E_{\mathrm{ab}}^{*}$ for calculating the Euclidean distance in CIELAB color space, the calculation formula is the same as Equation 8; (4) The calculation of CIEDE2000 color difference is Δ*E*_00_ and similar to that of LAB color difference, but some improvements based on subjective visual perception experiments are carried out to establish a linear relationship between the changes of lightness, hue, saturation, and visual perception, which is considered to be the best uniform color difference model in line with subjective visual perception, and its calculation formula is as Equation (22).


(23) $$\Delta \,E_{00}=\sqrt{{\left(\frac{\Delta \,L^{\prime }}{k_{L}\,S_{L}}\right)}^{2}+{\left(\frac{\Delta \,C^{\prime }}{k_{C}\,S_{C}}\right)}^{2}+{\left(\frac{\Delta \,H^{\prime }}{k_{H}\,S_{H}}\right)}^{2}+R_{T}\,\frac{\Delta \,C^{\prime }}{k_{C}\,S_{C}}\,\frac{\Delta \,H^{\prime }}{k_{H}\,S_{H}}}$$

*k*_*L*_, *k*_*C*_, *k*_*H*_ are environment-related correction factors, where *k*_*L*_ = *k*_*C*_ = *k*_*H*_ = 1. The color perception differences Δ*L*′,Δ*H*′ and Δ*C*′ are calculated in the same way as Equations (10), (11), and (12). *S*_*L*_, *S*_*C*_, and *S*_*H*_ are the luminance, chromaticity, and hue weighting factors, and *R*_*T*_ is the rotation factor used to correct for chromaticity differences to match visual perception (Yang et al., 2022). The smaller values of $\Delta \,E_{\mathrm{ab}}^{*}$ and Δ*E*_00_ indicate the smaller color perception differences of the samples.

## Results and discussion

First, we compared and analyzed the linearity of the mobile phone camera raw response. Then, spectral estimation experiments were conducted using raw responses from a variety of cell phone cameras, and professional DSLR cameras were used as comparisons to analyze whether they could achieve about the same estimation accuracy. Most importantly, the proposed method is compared with seven existing methods in experiments using the same sample data, and the error results and distributions are compared and analyzed in detail to demonstrate the superiority of the method. Finally, a color perception features weighting strategy is applied to the existing methods to demonstrate its positive effects.

### Verification of cell phone camera raw response

#### Linear test of raw response

**FIGURE 5:**
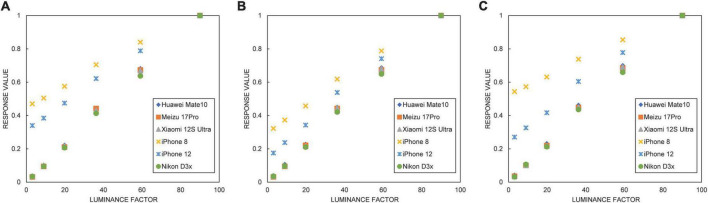
Linearity of the three channels of raw response for six imaging devices: **(A)** R channel, **(B)** G channel, and **(C)** B channel.

For full validation, several brands of cell phones with different operating systems and price ranges were used for comparison, and the list of devices is presented in Table 1. The Xrite ColorChecker CLASSIC color charts were photographed under the same light source conditions, and the grayscale response color blocks of CC charts were checked for their linearity using the raw response extraction method described in “Imaging conditions and raw response extraction,” as shown in Figure 5.

The horizontal coordinate in Figure 5 is the luminance factor of the XYZ color space of the grayscale color block of the CC color card, and the vertical coordinate is the three-channel value of the raw response. Figure 5 shows that the response linearity of the three Android phones is the same as that of the Nikon D3x. The response of the iPhone 8 and iPhone 12 also has good linearity, but the slope of linearity is a little smaller than that of the other four models. Overall, the raw response linearity of all five experimental phones is relatively good.

Further, we added JPEG photos from six devices for comparison, we present the *R*-squared values of the linear fitting of each channel of each device in Table 2, and the data show that the camera ISP post-processing significantly reduces the three-channel linearity of the JPG pictures. and it can be seen that the *R*-squared values of TIFF images of all devices are above 99%, while the *R*-squared values of JPG images are significantly lower. The above results show that the cell phone camera raw response itself has good linear performance, but after processing by the built-in chip and algorithm of the cell phone, the linearity of the three-channel response values of JPG images changes significantly and is difficult to simulate or characterize accurately, so the data source using raw response for spectral estimation is a better choice than images in formats such as jpg.

**TABLE 2:** *R*-squared values for each channel of TIFF and JPG for six imaging devices.

	R channel		G channel		B channel	
			
Device name	TIFF	JPG	TIFF	JPG	TIFF	JPG
Huawei mate10	0.998	0.776	0.998	0.766	0.996	0.754
Meizu 17pro	0.998	0.853	0.998	0.841	0.997	0.834
Xiaomi 12s ultra	0.999	0.877	0.998	0.872	0.997	0.870
iPhone 8	0.995	0.950	0.996	0.942	0.997	0.936
iPhone 12	0.997	0.843	0.996	0.822	0.993	0.815
Nikon d3x	0.999	0.850	0.999	0.843	0.998	0.838

#### Mobile phone raw response spectral estimation performance validation

Due to the extremely small internal space of cell phones, the CMOS sensor size is also several times smaller than that of DSLR cameras, and there is a huge difference between the imaging capability of cell phones and professional digital cameras, the impact of these factors on the spectral estimation accuracy has not been studied and verified. To verify the difference between the two raw responses, we still use the above six imaging devices and the same way to shoot SG color charts under the same illumination environment, with their 140 color blocks as the sample set, and use both the classical OLS method and the improved ALWLR method. To verify the relationship between the imaging capability and the spectral estimation accuracy of different cell phones, the Nikon D3x was also used as a comparison benchmark to evaluate the spectral estimation performance of each cell phone. The above experiments still use ten times ten-fold cross-validated to improve the stability of the results, and the experimental results are shown in Table 3.

**TABLE 3:** Performance comparison of raw responses in OLS method and ALWLR method for six devices: **(A)** RMSE, **(B)** GFC, **(C)** $\Delta \,E_{\mathrm{ab}}^{*}$, and **(D)** Δ*E*_00_.

(A)								

Device	OLS				ALWLR			
		
	Mean	Max	Med	p80	Mean	Max	Med	p80
Huawei mate10	4.804	12.573	3.892	5.277	2.784	**7.908**	2.302	3.940
Meizu 17pro	4.790	**11.231**	4.028	5.584	3.163	9.436	2.390	4.513
Xiaomi 12s ultra	**4.636**	12.423	**3.714**	**5.276**	2.938	8.518	2.360	4.088
iPhone 8	10.226	23.187	8.942	12.996	3.004	8.854	2.325	4.253
iPhone 12	8.830	18.212	8.183	11.212	2.977	8.898	2.353	4.131
Nikon d3x	5.000	12.712	4.255	5.496	**2.756**	8.477	**2.087**	**3.868**

**(B)**								

**Device**	**OLS**				**ALWLR**			
		
	**Mean**	**Max**	**Med**	**p80**	**Mean**	**Max**	**Med**	**p80**

Huawei mate10	98.705	99.944	99.439	98.589	**99.185**	99.994	99.674	**99.203**
Meizu 17pro	**98.835**	99.962	**99.557**	**98.743**	99.131	99.990	99.723	99.056
Xiaomi 12s ultra	98.784	**99.965**	99.459	98.638	99.123	99.988	99.685	99.059
iPhone 8	96.841	99.845	98.328	94.789	99.144	**99.996**	99.729	99.066
iPhone 12	96.785	99.853	98.459	94.701	99.100	99.991	99.716	99.047
Nikon d3x	98.134	99.964	99.312	97.221	99.112	99.991	**99.748**	99.039

**(C)**								

**Device**	**OLS**				**ALWLR**			
		
	**Mean**	**Max**	**Med**	**p80**	**Mean**	**Max**	**Med**	**p80**

Huawei mate10	3.956	9.814	3.006	**5.419**	1.408	2.857	1.345	1.937
Meizu 17pro	3.823	**9.429**	2.949	5.421	1.464	3.206	1.350	1.943
Xiaomi 12s ultra	**3.745**	9.857	**2.651**	5.456	1.384	3.184	1.244	1.905
iPhone 8	12.607	28.510	10.855	17.500	1.390	2.806	1.325	1.814
iPhone 12	11.502	25.330	9.920	16.215	1.284	**2.732**	1.200	1.761
Nikon d3x	4.597	13.444	3.147	6.631	**1.139**	2.881	**0.991**	**1.487**

**(D)**								

**Device**	**OLS**				**ALWLR**			
		
	**Mean**	**Max**	**Med**	**p80**	**Mean**	**Max**	**Med**	**p80**

Huawei mate10	5.440	**12.813**	4.098	7.740	2.062	5.209	1.757	2.793
Meizu 17pro	5.469	14.634	3.979	**7.538**	2.134	5.601	1.629	2.943
Xiaomi 12s ultra	**5.290**	13.582	**3.826**	7.762	2.026	5.741	1.615	2.754
iPhone 8	18.961	70.409	12.750	23.881	2.003	**4.942**	1.641	2.758
iPhone 12	16.226	49.534	11.726	23.193	1.913	5.407	1.490	2.647
Nikon d3x	6.471	17.885	4.531	9.520	**1.718**	5.024	**1.315**	**2.399**

For the OLS method, the accuracy of all indicators of the three Android phones is better than the D3x camera, with the Xiaomi 12s ultra having a relatively better performance in the 3 areas of RMSE, $\Delta \,E_{\mathrm{ab}}^{*}$. and Δ*E*_00_ errors; While the errors for two iPhones only had GFC errors close to the other four devices, the other three errors are all more than two times larger than the Android phone errors. For the ALWLR method, the situation changed because the samples were weighted and selected by color difference, which led to a substantial improvement in the estimation accuracy of all six devices, with a much smaller range of error between devices. In terms of RMSE error, Nikon D3x obtained the best overall estimation accuracy, with only the maximum value of RMSE higher than Huawei mate10, and the two iPhones are also very close to each other in the two items of mean and median RMSE. In terms of GFC indicators, the average value and the p80 error of Huawei mate10 were better than D3x, and the performance of the three Android phones was comparable, with Huawei mate10 being the best and slightly better than D3x, Meizu 17pro, and Xiaomi 12s ultra having the average value and the p80 GFC better than D3x, so three Android phones were better than Nikon D3x in terms of GFC mean and p80 error. The GFC errors of the 2 Apple phones were also at the same level. The chromaticity error of $\Delta \,E_{\mathrm{ab}}^{*}$ and Δ*E*_00_ Nikon D3x performance is slightly better, the difference between the color difference value of these six device were very small, the $\Delta \,E_{\mathrm{ab}}^{*}$ mean value range is within 0.4, and the range of mean value of Δ*E*_00_ is within 0.45.

In general, in the classical spectral estimation methods like OLS, Android phones are better than professional cameras in all four metrics. For the ALWLR method, after effective sample selection and weighting using color difference, the spectral estimation errors of all devices are significantly reduced, and the p80 of each error is smaller than the mean error of the OLS method. The mean value of the single error of some cell phones is better than that of professional cameras, while other indicators have a small difference with professional digital cameras, and the chromaticity error is much smaller than the discriminatory ability of the human eye.

The results show that, under the same conditions, the influence of the estimation method on the estimation accuracy is decisive, while under the more complex methods, the difference in estimation accuracy between most cell phone camera raw responses and professional digital cameras is very small, while some evaluation indicators are better than professional cameras. Therefore, we believe that it is feasible for cell phones to replace professional digital cameras as spectral imaging devices overall.

### Comparison of methods

In this section, the number of polynomial expansion items of the proposed method is first experimented to determine the optimal number of expansion terms. Then the proposed method is compared and analyzed together with representative existing methods. The methods that participated in the comparison mainly include Ordinary least squares (OLS) (Connah and Hardeberg, 2005), Partial least squares (PLS) (Shen et al., 2010), PCA (Xiao et al., 2016), Local linear weighting method (LLR) (Liang and Wan, 2017), Weighted non-linear regression method (WNR) (Amiri and Amirshahi, 2014), the local adaptive weighting method (ALWLR) (Liang et al., 2019), and the sequential adaptive weighted non-linear regression method (SWNR) (Wang et al., 2020). In addition, the running time of the eight methods was also counted and compared.

#### Influence of the number of polynomial expansion items on the proposed method

As described in the principle of the method in section “The proposed method,” a polynomial expansion of the camera raw response is performed, so the appropriate number of expansion items needs to be determined, and we experimented with the performance of the proposed method by ranging the number of expansion items from 4 to 35, with the sample set of SG color chart taken from the well-balanced Xiaomi 12S Ultra in section “Mobile phone raw response spectral estimation performance validation” and the same light source environment as described before. The effect of the change in the number of items of the polynomial expansion on the spectral estimation error of this method is shown in Table 4.

**TABLE 4:** The estimation accuracy of the proposed method spectral estimation method with the number of polynomial expansions from 4 to 35 items.

Items	RMSE			GFC			$\Delta \,E_{\mathrm{ab}}^{*}$			Δ*E*_00_		
				
	Mean	Med	p80	Mean	med	p80	Mean	Med	p80	Mean	Med	p80
4	2.493	1.769	3.444	99.484	99.902	99.550	1.051	0.938	1.356	1.651	1.282	2.207
5	2.311	1.578	3.164	99.502	99.895	99.562	1.005	0.897	1.292	1.567	1.190	2.075
6	2.256	1.559	3.018	99.517	99.898	99.598	0.966	0.851	1.253	1.498	1.135	1.978
7	2.239	1.522	3.060	99.522	99.910	99.621	0.917	0.791	1.230	1.444	1.056	1.976
8	2.087	1.427	**2.793**	99.541	99.920	99.666	0.881	0.759	1.143	1.374	1.007	1.808
9	2.025	1.454	2.825	99.584	99.928	99.669	0.862	0.764	1.146	1.331	1.000	1.830
10	2.019	1.467	2.865	99.583	99.919	99.659	0.858	0.758	1.152	1.299	1.017	1.756
11	**2.000**	1.432	2.885	**99.588**	99.925	99.675	0.847	0.739	**1.122**	1.281	0.986	**1.731**
12	2.029	1.460	2.863	99.583	99.928	99.671	0.868	0.740	1.171	1.311	1.008	1.804
13	2.007	1.430	2.863	99.579	99.932	99.655	0.854	0.725	1.152	1.301	0.985	1.790
14	2.040	1.457	2.869	99.576	99.926	99.661	0.857	0.727	1.153	1.309	**0.972**	1.818
15	2.025	1.444	2.855	99.572	99.930	99.663	0.857	0.738	1.162	1.314	0.990	1.804
16	2.026	1.401	2.930	99.567	99.927	99.636	0.840	0.723	1.142	1.274	0.977	1.797
17	2.042	1.421	2.911	99.558	99.936	99.655	0.847	0.729	1.158	1.309	1.012	1.815
18	2.013	1.382	2.886	99.572	99.935	99.669	**0.826**	0.716	1.126	1.288	0.975	1.768
19	2.040	1.397	2.864	99.573	99.931	**99.679**	0.838	0.717	1.143	**1.268**	0.978	1.812
20	2.083	1.429	2.940	99.563	99.934	99.642	0.849	0.739	1.164	1.301	0.999	1.865
21	2.070	1.409	2.921	99.556	99.934	99.625	0.837	0.715	1.149	1.281	0.984	1.838
22	2.037	1.425	2.870	99.551	99.933	99.670	0.866	0.709	1.188	1.321	0.994	1.901
23	2.032	1.389	2.913	99.563	99.933	99.661	0.878	**0.691**	1.206	1.417	0.966	1.954
24	2.047	1.386	2.877	99.572	**99.939**	99.672	0.912	0.723	1.241	1.463	1.003	1.960
25	2.120	1.396	2.969	99.536	**99.939**	99.659	0.935	0.698	1.271	1.618	0.994	2.029
26	2.228	**1.363**	3.043	99.510	99.934	99.647	1.004	0.693	1.266	2.040	0.985	2.029
27	2.287	1.479	3.163	99.509	99.923	99.594	0.998	0.727	1.316	1.826	1.046	2.157
28	2.341	1.432	3.137	99.487	99.921	99.600	1.020	0.720	1.304	1.934	1.024	2.152
29	2.417	1.457	3.242	99.452	99.929	99.568	1.037	0.735	1.321	1.952	1.056	2.127
30	2.377	1.447	3.213	99.434	99.926	99.606	0.987	0.745	1.326	1.672	1.061	2.157
31	2.474	1.481	3.452	99.480	99.912	99.560	1.030	0.762	1.356	1.716	1.100	2.258
32	2.433	1.497	3.398	99.482	99.916	99.538	1.034	0.764	1.407	1.717	1.073	2.321
33	2.544	1.527	3.547	99.391	99.911	99.485	1.070	0.774	1.469	1.838	1.106	2.403
34	2.621	1.495	3.644	99.330	99.908	99.527	1.093	0.796	1.486	1.834	1.137	2.507
35	2.731	1.637	3.945	99.281	99.893	99.451	1.161	0.837	1.585	2.150	1.212	2.659

The bold values indicate the best estimation accuracy.

The data in Table 4 show that the mean, median, and p80 errors of the four indicators have the same trend, decreasing and then increasing as the number of items increases. In the interval of 9–22 items, it is obvious that each error is in the optimal interval and the differences is very small. As the number of terms exceeds 22, the accuracy of each estimate decreases gradually. After comparison, we choose 18 items with relatively more balanced errors as the number of polynomial expansion items for the subsequent study.

#### Comparison of estimation methods

We compare the proposed methods with seven existing methods in detail, where the WNR, ALWLR, and SANR methods contain polynomial expansions and the number of terms we fix to 18. Some of the methods include the selection of samples, and we fix the local sample selection parameter L to be 100 for all methods, which means that the first 100 local samples are selected. All other aspects of the experiments were kept consistent and used the same sample set of SG color chart taken by Xiaomi 12S Ultra.

Ten times 10-fold cross-validation was used for all eight methods to enhance the stability of the resulting data. The estimated results are shown in Table 5.

**TABLE 5:** Comparison of estimation accuracy between the proposed method and seven existing methods: **(A)** RMSE, **(B)** GFC, **(C)** $\Delta \,E_{\mathrm{ab}}^{*}$, and **(D)** Δ*E*_00_.

(A)								

Error Type	WNR	LLR	ALWLR	OLS	PCA	PLS	Proposed	SWNR
Mean	2.582	2.122	2.946	4.641	6.701	3.834	**1.982**	2.648
Max	10.731	7.510	8.482	11.824	12.733	10.574	**7.216**	9.282
Med	1.618	1.533	2.360	3.767	6.354	3.065	**1.373**	1.767
Min	0.217	0.108	0.320	2.028	2.306	1.439	**0.063**	0.145
p80	3.270	2.926	4.236	5.227	9.178	4.634	**2.788**	3.414

**(B)**								

**Error Type**	**WNR**	**LLR**	**ALWLR**	**OLS**	**PCA**	**PLS**	**Proposed**	**SWNR**

Mean	99.381	99.399	99.141	98.781	95.424	98.674	**99.552**	99.425
Max	99.999	**100.000**	99.992	99.971	99.954	99.970	**100.000**	99.998
Med	99.882	99.911	99.683	99.429	98.838	99.368	**99.937**	99.921
Min	95.169	94.785	95.018	93.525	80.248	94.580	**96.102**	95.520
p80	99.507	99.583	99.116	98.557	92.665	97.936	**99.704**	99.636

**(C)**								

**Error Type**	**WNR**	**LLR**	**ALWLR**	**OLS**	**PCA**	**PLS**	**Proposed**	**SWNR**

Mean	1.184	0.906	1.385	3.746	9.856	1.844	**0.801**	1.185
Max	3.401	2.244	3.171	9.596	24.557	3.529	**2.103**	2.964
Med	1.037	0.761	1.233	2.703	7.095	1.717	**0.675**	1.010
Min	0.154	0.187	0.268	0.782	1.698	0.758	**0.116**	0.313
p80	1.510	1.243	1.883	5.601	15.341	2.336	**1.118**	1.406

**(D)**								

**Error Type**	**WNR**	**LLR**	**ALWLR**	**OLS**	**PCA**	**PLS**	**Proposed**	**SWNR**

Mean	1.725	1.381	2.036	5.295	16.197	2.532	**1.250**	1.671
Max	5.764	4.313	5.876	13.229	43.722	6.519	**3.658**	5.155
Med	1.262	1.002	1.620	3.906	10.821	2.127	**0.964**	1.258
Min	0.205	0.190	0.290	1.013	2.279	1.034	**0.130**	0.375
p80	2.146	1.900	2.793	8.078	24.929	2.905	**1.798**	2.032

The bold values indicate the best estimation accuracy.

**FIGURE 6:**
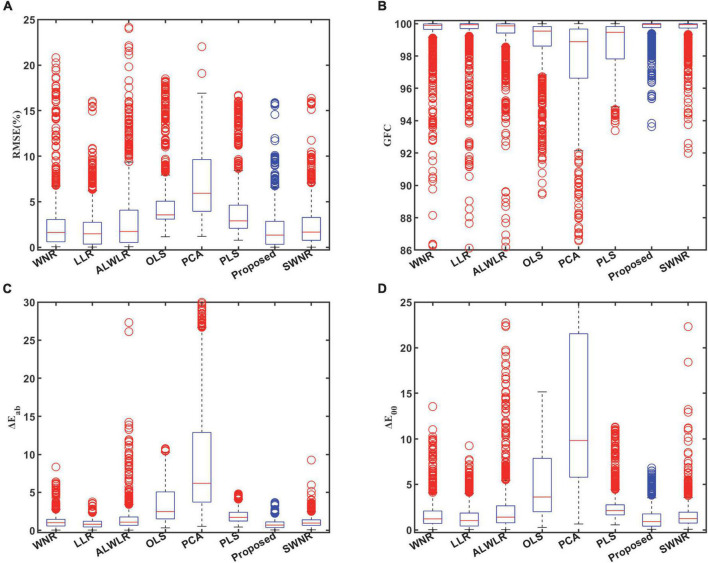
Boxplots of estimation errors for the comparison of eight methods: **(A)** RMSE, **(B)** GFC, **(C)** $\Delta \,E_{\mathrm{ab}}^{*}$, and **(D)** Δ*E*_00_.

In Table 5 we bold the data with the best accuracy in each row, and it can be seen that the proposed method in this paper obtains the best spectral reflectance estimation performance in each of the four estimated accuracy metrics. From the data in Table 5. We can also observe that the OLS, PCA, and PLS methods have greater overall errors, while the improved variants such as WNR, LLR, ALWLR, and SWNR methods have better estimation accuracy performance and less difference from the proposed method due to different forms of weighting and sample selection. To further analyze the method estimation error performance, we plotted boxplots for the four evaluation indicators of the above eight methods, as in Figure 6.

From the four boxplots in Figure 6, it can be seen that the distance between the upper and lower edges of the error distribution and the height of the box of the proposed method are more intensive, while the distance of the box from the minimum value is the closest in all four evaluation indexes, showing that the present method has a smaller overall error. Also, the outlier data of this method are more concentrated near the upper edge of the normal value (GFC is the lower edge).

#### Comparison of method running efficiency

We counted the running time of each method. All methods count the computation time of 10 times 10-fold cross-validation (excluding the time to save the results) as shown in Table 6, and we counted the time of five runs of 10 times 10-fold cross-validation to get the average time. The hardware is Lenovo Legion R9000P2021H laptop, CPU is AMD Ryzen7 5800H 3.2GHz with 8 cores and 16 threads, memory is 16GB DDR4-3200MHz, the software is MATLAB R2021a, the operating system is 64-bit windows 10. It can be seen that the time of the OLS and PLS methods without weighting and selection of samples for ten times ten-fold cross-validation is within 1 s, and the computing time of the WNR, LLR, and ALWLR methods with one weighting and selection of samples is about more than 1.5 s, and the proposed method increases by 0.5 s compared with the SANR method with the same two weightings because of the addition of three color perception feature volume calculations, and all The average time of all methods is within 2 s, which means that the difference in running time is very small even on a home computer.

**TABLE 6:** Five running time of 10 times 10-fold cross-validation for eight methods and the average.

Method	First	Second	Third	Fourth	Fifth	Average time(s)
WNR	1.222	1.196	1.211	1.247	1.245	1.224
LLR	1.237	1.226	1.213	1.242	1.232	1.230
ALWLR	1.226	1.204	1.229	1.219	1.260	1.228
OLS	0.953	0.951	0.935	0.945	0.934	0.943
PCA	5.020	5.018	4.900	4.967	4.876	4.956
PLS	0.930	0.941	0.933	0.944	0.918	0.933
Proposed	2.005	1.991	1.948	1.975	1.989	1.981
SWNR	1.551	1.516	1.609	1.582	1.559	1.564

According to the comparison and analysis of the above three aspects, the proposed method is ahead of the existing methods in all aspects of estimation accuracy, and the overall error is smaller and the error distribution is more intensive, while the time cost is slightly increased than the existing methods, but the difference is not significant, which proves the superiority of the proposed method.

### Application of color perception features weighting strategy on existing methods

The novelty of this method is to propose a strategy of weighting color perceptual features based on CIELAB uniform color space, which calculates the differences of color perceptual features of samples in LAB color space instead of color difference weighting and achieves better performance. In this section, we try to apply the weighting strategy proposed to the OLS method, the WNR method, and the ALWLR method. The sample set still uses the SG color chart taken by Xiaomi 12S Ultra. The estimated accuracies of the above four methods before and after applying the proposed weighting strategy are presented in Table 7. The methods containing the ‘opt-’ prefix are the methods after applying the proposed weighting strategy.

**TABLE 7:** Comparison of the estimation accuracy of four methods applying the weighting strategy: **(A)** RMSE, **(B)** GFC, **(C)** $\Delta \,E_{\mathrm{ab}}^{*}$, and **(D)** Δ*E*_00_.

(A)								

Error Type	WNR	LLR	ALWLR	OLS	opt-WNR	opt-LLR	opt-ALWLR	opt-OLS
Mean	2.648	2.046	2.979	4.643	2.056	2.094	**2.043**	2.461
Max	11.355	**7.272**	8.773	11.908	7.595	7.800	7.567	7.867
Median	1.662	1.508	2.361	3.739	**1.401**	1.423	1.536	1.949
Min	0.222	0.121	0.376	1.990	0.089	**0.076**	0.183	0.150
p80	3.321	2.857	4.103	5.241	2.841	2.914	**2.564**	3.415

**(B)**								

**Error type**	**WNR**	**LLR**	**ALWLR**	**OLS**	**opt-WNR**	**opt-LLR**	**opt-ALWLR**	**opt-OLS**

Mean	99.3661	99.4465	99.0919	98.7684	99.4967	99.4681	**99.5646**	99.4520
Max	99.9992	99.9996	99.9903	99.9628	**99.9999**	**99.9999**	99.9994	99.9998
Median	99.8750	99.9206	99.6700	99.4620	99.9363	99.9297	**99.9381**	99.8967
Min	94.8655	95.3612	94.7806	93.7069	95.7146	95.5170	**96.5514**	95.9500
p80	99.5692	99.5902	98.9699	98.5126	**99.6699**	99.6536	99.6605	99.4762

**(C)**								

**Error type**	**WNR**	**LLR**	**ALWLR**	**OLS**	**opt-WNR**	**opt-LLR**	**opt-ALWLR**	**opt-OLS**

Mean	1.189	0.893	1.392	3.743	**0.840**	0.846	0.949	1.318
Max	3.466	2.227	3.226	9.843	2.114	2.148	**2.095**	4.416
Median	1.029	0.766	1.224	2.629	0.735	**0.706**	0.874	0.919
Min	0.166	0.191	0.287	0.786	0.168	**0.147**	0.270	0.238
p80	1.504	1.215	1.906	5.648	1.163	**1.160**	1.228	1.668

**(D)**								

**Error type**	**WNR**	**LLR**	**ALWLR**	**OLS**	**opt-WNR**	**opt-LLR**	**opt-ALWLR**	**opt-OLS**

Mean	1.747	1.350	2.053	5.286	1.332	**1.328**	1.383	2.003
Max	5.963	4.325	5.903	13.458	4.112	3.934	**4.079**	6.458
Median	1.265	1.041	1.649	3.865	**1.007**	1.023	1.116	1.374
Min	0.216	0.200	0.311	1.021	**0.166**	0.149	0.325	0.253
p80	2.256	1.808	2.783	8.040	1.856	1.877	**1.800**	2.831

The bold values indicate the best estimation accuracy.

From the overall view of the data in Table 7, all four methods obtained a reduction in estimation error after applying this weighting strategy. Among them, opt-OLS has a greater improvement due to its relatively larger original error. The errors of opt-WNR, opt-LLR, and opt-ALWLR methods were also significantly reduced relative to the original methods in four aspects, for example, in the GFC data in sub-table (B), the 80% error of these three methods exceeded 99.6%; In sub-table (C) $\Delta \,E_{\mathrm{ab}}^{*}$, all three methods, opt-WNR, opt-LLR, and opt-ALWLR, were reduced to within 1 in the mean and median values.

In summary, for OLS methods without sample weighting and selection, the application of the present weighting strategy brings a significant improvement. For the methods such as WNR, LLR, and ALWLR that have been optimized with sample weighting and selection, the errors in each spectral and chromaticity are also noticeably reduced. The good improvement of the proposed weighting strategy is proved.

### Discussion

The proposed method uses the idea of two-times sample weighting and selection similar to the SWNR method. By improving the weighting method of the first spectral estimation and using the weighting method of color perception features in CIELAB color space instead of the weighting method based on color difference or based on chromaticity vector angle, a more accurate measure of color difference between samples and a better estimation accuracy are achieved.

The color perceptual features of CIELAB uniform color space include hue, chroma, and lightness, which are closely related to the visual perception of human eyes. The weights are directly related to these three perceptual features in our weighting approach, avoiding the problem of being given the same weight because of the equal color difference Euclidean distance, and thus being more effective than the weighting approach of existing methods.

At the same time, this study also has the following limitations: (1) The color sample sets for the experiments all use the samples of 140 color blocks of the SG color chart, and further adaptation experiments are needed on other sample sets. (2) The validation of the method is conducted under the same light source conditions, and the stability of the method needs to be studied under more light source conditions.

## Conclusion

This study starts with the cell phone camera raw response. First, the linearity verification and spectral estimation performance of the cell phone camera raw response are studied, and it is confirmed that it has good linearity at the same time, and the spectral estimation performance is comparable to the professional digital cameras under the same conditions, which proves the feasibility of using the cell phone camera raw response for spectral estimation. Then a sequence adaptive weighted optimal spectral estimation method based on color perception features is proposed, and the effect of polynomial expansion on the proposed method is investigated to obtain the optimal number of expansion items. Most importantly, a detailed comparison experiment based on the raw response of the cell phone camera with the existing methods shows that the proposed method has the best performance in all four metrics of spectral error and chromaticity error. Further, we apply the weighting strategy based on color perception features to the existing method, and the comparison results show that the estimation accuracy is improved after applying the strategy, which fully demonstrates the superiority of the method and the excellent effect of the weighting strategy on the estimation error reduction.

In the future, further research is needed for cell phone camera imaging characterization; based on more different types of color sample sets, spectral estimation methods need to be improved to enhance their generalization capabilities. Spectral estimation methods based on cell phone cameras under different light source conditions also need to be further studied.

## Data availability statement

The original contributions presented in this study are included in the article/supplementary material, further inquiries can be directed to the corresponding author/s.

## Author contributions

DL: conceptualization, methodology, software, investigation, formal analysis, writing – original draft, writing – review and editing, and visualization. XWW: data curation, formal analysis, and writing – review and editing. JL: conceptualization, writing – review and editing. TW: writing – review and editing. XXW: resources, supervision, writing – review and editing. All authors contributed to the article and approved the submitted version.

## Footnotes

1 DNG format is a publicly available archival format for raw files. Details in: https://helpx.adobe.com/camera-raw/digital-negative.html.

2 NEF format is the RAW format of Nikon DSLR Camera. Details in: https://imaging.nikon.com/lineup/dslr/basics/26/01.htm.
